# Evaluating the efficacy of vatiquinone in preclinical models of mitochondrial disease

**DOI:** 10.21203/rs.3.rs-4202689/v1

**Published:** 2024-06-03

**Authors:** Ernst-Bernhard Kayser, Yihan Chen, Michael Mulholland, Vivian Truong, Katerina James, Allison Hanaford, Simon Johnson

**Affiliations:** Seattle Children’s Research Institute; Seattle Children’s Research Institute; Northumbria University; Northumbria University; Northumbria University; Seattle Children’s Research Institute; Northumbria University

**Keywords:** Mitochondrial disease, antioxidant, vatiquinone, EPI-743, ROS, Leigh syndrome, GPX4

## Abstract

**Background:**

Genetic mitochondrial diseases are a major challenge in modern medicine, impacting around 1:4,000 individuals. Leigh syndrome is the most common pediatric presentation of mitochondrial disease. There are currently no effective clinical treatments for mitochondrial disease. In humans, patients are often treated with antioxidants, vitamins, and strategies targeting energetics. The vitamin-E related compound vatiquinone (EPI-743, α-tocotrienol quinone) has been the subject of at least 19 clinical trials in the US since 2012, but the effects of vatiquinone on an animal model of mitochondrial disease have not yet been reported. Here, assessed the impact of vatiquinone on disease progression and in two animal models of mitochondrial disease.

**Methods:**

The efficacy of vatiquinone *in vitro* was assessed using human fibroblasts treated with the general mitochondrial oxidative stress inducer paraquat, the GPX4 inhibitor RSL3, or the glutathione synthase inhibitor BSO in combination with excess iron. The therapeutic potential of vatiquinone *in vivo* was assessed using tamoxifen-induced mouse model for GPX4 deficiency and the *Ndufs4* knockout mouse model of Leigh syndrome. In both models, animals were treated daily with vatiquinone or vehicle and relevant disease endpoints were assessed.

**Results:**

Vatiquinone robustly prevented death in cultured cells induced by RSL3 or BSO/iron, but had no effect on paraquat induced cell death. Vatiquinone had no impact on disease onset, progression, or survival in either the tamoxifen-inducible GPX4 deficient model or the *Ndufs4*(−/−) mouse model, though the drug may have reduced seizure risk.

**Conclusions:**

Vatiquinone provided no benefit to survival in two mouse models of disease, but may prevent seizures in the *Ndufs4*(−/−) model. Our findings are consistent with recent press statements regarding clinical trial results and have implications for drug trial design and reporting in patients with rare diseases.

## Introduction

Mitochondrial diseases, disorders caused by defects in genes encoding mitochondrial factors, are a major challenge in human medicine. As a group, mitochondrial diseases are the most common cause of inborn errors of metabolism, and a major cause of genetic neurologic disease. Mitochondrial diseases are estimated to impact approximately 1 in 4,000 individuals, though this may underestimate actual cases due to challenges in diagnosis.

Mitochondrial diseases are a genetically and clinically diverse group of disorders; over 300 unique genetic defects have been shown to cause mitochondrial disease. Mitochondrial disease can be caused by defects in genes that directly impact mitochondrial electron transport chain (ETC) subunits, genes encoding products peripherally involved in ETC function, or genes involved in other mitochondrial processes such as antioxidant defenses. Friedreich’s ataxia (FA), for example, is caused by defects in frataxin, a mitochondria-localized protein involved in iron homeostasis, while deficiencies in the mitochondria-localized lipid antioxidant enzyme GPX4 can cause Sedaghatian-type spinal metaphyseal dysplasia (1–3).

Over a dozen distinct named clinical syndromes can result from genetic mitochondrial dysfunction. These can involve single organ dysfunction, as in optic nerve pathology in Leber’s Hereditary Optic Neuropathy (LHON), or occur as complex multi-system disorders such as Leigh syndrome (4, 5). Leigh syndrome, or subacute necrotizing encephalopathy, is one of the more severe clinical presentations of mitochondrial disease and the most common pediatric presentation. Leigh syndrome is characterized by early post-natal onset (median ~ 2 years, though adult onset can occur) with muscle weakness, failure to thrive, metabolic dysfunction including hyperlactatemia, and symmetric progressive necrotizing lesions in brain areas including the basal ganglion, and seizures. The CNS lesions are a defining feature of the disease, drive severe neurologic defects, and typically lead to mortality from respiratory failure. Leigh syndrome is genetically heterogeneous: defects in over 100 unique genes in the nuclear and mitochondrial genomes have been identified as causes of Leigh syndrome (5).

Clinically effective treatments do not yet exist for genetic mitochondrial diseases of any etiology. Disease management is generally limited to treating symptoms and providing supportive care. Patients are often also given vitamins, antioxidants, and/or nutritional supplements, often provided in combination as the ‘mito-cocktail’(6). Though likely benign, these strategies likely provide little or no benefit in most forms of mitochondrial disease. In fact, in the majority of cases, it is unclear whether oxidative damage or energetics play any direct causal role in mitochondrial disease pathogenesis. Nonetheless, drugs targeting oxidative stress and energetics have been the subject of multiple clinical trials in mitochondrial disease.

Vatiquinone, previously known as EPI-743, is a vitamin E derived compound under development by PTC Therapeutics, acquired from BioElectron Technology Corporation (formerly known of as Edison Pharmaceuticals) in 2019. Vatiquinone robustly suppresses cell death induced by certain forms of redox stress in cultured cells (7). At least 19 human trials of vatiquinone have been initiated, mainly targeting populations with genetic mitochondrial diseases including Leigh syndrome, FA, LHON, and mitochondrial disease as a group (see Table 1, Discussion). Cell-based endpoints provided the rationale for these trials, but no studies have directly assessed the potential efficacy of vatiquinone *in vivo* using an animal model. Given the limited mechanistic overlap between cultured cell toxicity/viability assays and mitochondrial disease pathobiology *in vivo*, we sought here to directly assess the efficacy of vatiquinone *in vivo* using animal models of genetic mitochondrial disease.

## Methods

### Vatiquinone

Vatiquinone was purchased commercially from MedKoo Biosciences (Morrisville, NC). At room temperature, vatiquinone is a viscous liquid. For cell culture studies, vatiquinone stock solutions were prepared by dissolving vatiquinone in DMSO. For animal treatments by IP injection, vatiquinone was dissolved in sterile sunflower seed oil or 95% sunflower seed oil with 5% ethanol when co-injected with tamoxifen (details below). In each case, stock solutions were aliquoted and stored at −20° C or −80° C, protected from light, until warmed for injection.

### Cultured Fibroblasts

Human dermal neonatal fibroblasts (Sigma 106-05N) were propagated in low-glucose DMEM (D5523) supplemented with 10% fetal bovine serum (FBS, FisherScientific UK 11570506), 100 U/mL penicillin & streptomycin (ThermoFisher 15140122), and D-glucose (Sigma 1083370250) added to a final concentration of 12.5mM. Cells were grown in 5% CO_2_ balanced with air at 37° C. Fibroblasts were maintained by splitting 1:4 when confluent, all experiments in fibroblasts were between population doubling numbers of 24–30.

### Cell Viability Assays

Human neonatal fibroblasts were plated onto 24-well plates 1:4 from plates that had just reached approximately 95% confluent and incubated for 48 hours for adherence and growth to approximately 50% confluence. These cells were exposed to conditions as detailed below (see paraquat, RSL3, BSO/Fe(III) Citrate). At the end of these exposures, media was removed from wells and replaced with pre-warmed 1X PBS (ThermoFisher 10010023, pH 7.4, containing Hoechst 33342 (Sigma 14533) and ethidium homodimer 1 (Ethd1, Sigma 46043) at 1µg/mL and 2µM, respectively. Cells were incubated in dye solution for 20 minutes at 37° C. After incubation, cells were imaged on a FLoid (Invitrogen 4471136) inverted fluorescent microscope equipped with brightfield, DAPI (excitation: 390/40 nm, emission 446/33 nm), GFP (excitation 482/18 nm, emission 532/59 nm), and RFP (excitation 586/15 nm, emission 646/68 nm) cubes. Cells staining positively for with Ethd-1 (a cell-impermeant DNA dye, RFP filter cube) were deemed non-viable, while Hoechst 33342 (a cell permeant DNA dye, DAPI filter cube) was used to identify all nuclei. In each assay, a vatiquinone stock solution was prepared by mixing vatiquinone (liquid), measured by weight, into DMSO to 400µM final concentration.

#### Paraquat:

Anhydrous paraquat was solubilized in 1X PBS to a final stock concentration of 1M. This diluted in culture media to working concentrations of 5 mM and 0.5 mM. Vatiquinone stock solutions (1,000X in DMSO, 125 µM and 500 µM) were diluted in full cell culture media to the appropriate wells to reach the desired concentration (125 nM or 500 nM). Equal DMSO added to non-vatiquinone wells. Viability was assessed (as outlined above) at 24, 48, 72, 96, and 144 hours following paraquat treatment.

#### RSL3:

Anhydrous RSL3 was solubilized in DMSO to generate a stock solution of 10 mM. Vatiquinone (or DMSO) containing media was added first; after 1 hour, media was replaced with media containing RSL3 or vehicle along with vatiquinone or DMSO, and cells were returned to the cell culture incubator. Viability was assessed (as outlined above) at 24 hours after addition of RSL3.

#### BSO/Fe(III)Citrate:

Ferric citrate (Fe(III)C) was dissolved in full cell culture media to a stock concentration of 2.5 mM. L-buthionine-(S,R)-sulfoximine (BSO) was dissolved in 1XPBS to a stock concentration of 200 µM. Fe(III)C and BSO were added to cell culture media to prepare media containing 25 µM BSO / 100 µM Fe(III)C. Vatiquione or vehicle (DMSO) was added to cells, they were incubated for one hour, then media was replaced with cell media containing 25 µM BSO / 100 µM Fe(III)C with vatiquinone or vehicle (DMSO). Viability was assessed at 48 hours following addition of BSO/Fe(III)C using the method detailed above.

### Ethics statement on animal use

All experiments were approved by the Institute Animal Care and Use Committee at Seattle Children’s Research Institute (Seattle, WA) under protocols IACUC00611 and IACUC00070. Experiments contain similar numbers of male and female mice of each genotype, numbers are provided in figure legends and supplemental files.

### General mouse husbandry

Mice were kept under standard vivarium conditions at Seattle Children’s Research Institute, with a 12-hour light/dark cycle, with the light phase occurring during the day. Mice were socially housed in Thoren cages with no limits to access to food and water. Chow was supplied ad libitum from hoppers. Cages with mice showing neurological symptoms received additional wetted chow and water in dishes on the bottom of the cage to ensure that food and water accessibility would not become a factor for disease progression and survival. All pups were weaned at age P20 or P21.

In experimental animals, the following health parameters were assessed a minimum of 3 times per week, and on every day when animals received an injection (see below):

#### Body weight/Cachexia:

**Figure 1:**
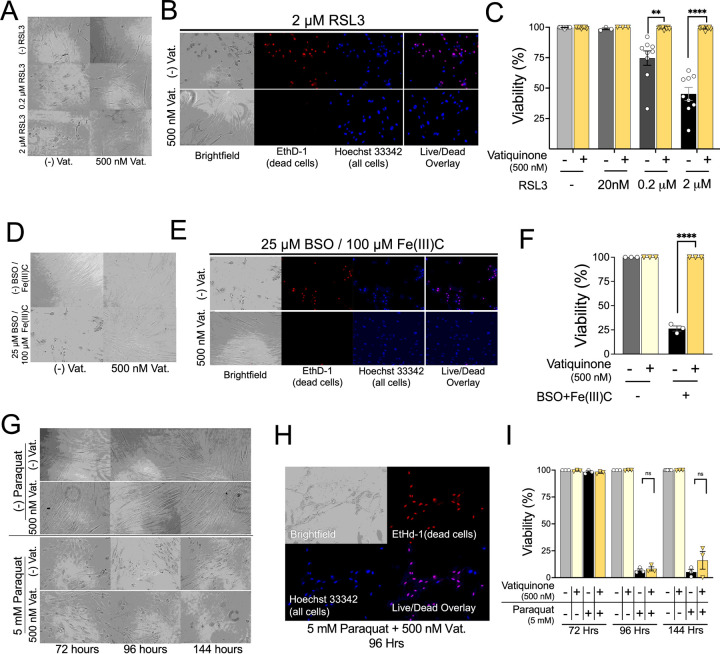
Vatiquinone prevents RSL3 and BSO/Fe(III)C, but not paraquat, induced cell death. (A-C) RLS toxicity assay. Given the relatively short time of this assay (24 hours), vatiquinone (or DMSO) was added one hour prior to addition of RSL3. (A) Brightfield images of cells (human dermal fibroblasts, HDFs, see Methods) at 24 hours of exposure to 0, 0.2 µM, or 2 µM RSL3 in the presence or absence of 500 nM vatiquinone. RSL3 exposure at both 0.2 µM and 2 µM induced significant morphologic changes by 24 hours which were prevented by vatiquinone. (B) Representative cell viability staining images from cells exposed to 2 µM RSL3 with or without 500 nM vatiquinone. EthD-1 – ethidium homodimer 1, a cell-impermeant dye that stains the nuclei of dead but not viable (plasma membrane intact) cells. Hoechst 33342 – cell-permeant DNA-binding dye, counterstain for all cells. (C) Cell viability at 24 hours in cells exposed to RSL3 with or without vatiquinone, as indicated. Viability defined as percent of cells (identified by Hoechst 33342) which were not stained with EthD-1. Vatiquinone fully suppressed cell death induced by 0.2 µM and 2 µM RSL3, consistent with the rescue of cell morphology. ** p<0.005, ****p<0.0001 by pairwise t-test. n=9 individual replicate wells per group, spread across three individual experiments. (D) Brightfield images of HDFs after 48 hours of exposure to control conditions or 25 µM BSO with 100 µM Fe(III)Citrate (Fe(III)C) in the presence or absence of 500 nM vatiquinone. BSO+Fe(III)C induced significant morphologic changes which were prevented by vatiquinone. (E) Representative cell viability staining (dyes as detailed in (B)) from cells treated with BSO and 25 µM BSO / 100 µM Fe(III)C in the presence or absence of 500 nM vatiquinone. (F) Cell viability at 24 hours in cells exposed to RSL3 with or without vatiquinone, as indicated. Vatiquinone fully suppressed cell death induced by 25 µM BSO / 100 µM Fe(III)C at 48 hours. ****p<0.0001 by pairwise t-test. n=3 replicates per treatment. (G) Brightfield images of fibroblasts after 72, 96, or 144 hours exposure to 5 mM paraquat in the presence or absence of 500 nM vatiquinone. Paraquat exposure at induced significant morphologic changes by 72 hours. Vatiquinone had no effect on these morphologic changes. (H) Representative cell viability staining images from cells exposed to paraquat and vatiquinone for 144 hours, dyes as detailed above. (I) Cell viability at 72, 96, and 144 hours in cells exposed to paraquat with or without vatiquinone, as indicated. Viability defined as fraction of nuclei (identified by Hoechst 33342) which were not stained with EthD-1. While marked morphologic changes occurred by 72 hours of paraquat exposure, significant cell death was not observed until 96 hours of exposure. Vatiquinone did not significantly impact cell death induced by paraquat. n.s. – not significant by pairwise t-test. n.s. – not significant. n=3 per timepoint in each treatment.

Cachexia onset (Figure 1 and Figure 3) is reported as the post-natal day when an individual animal’s weight peaks, i.e. when progressive weight loss starts, as in our prior studies (8–11).

*Forelimb clasping*, a sign of neurologic disease progression, was scored, as previously described (8–11). As disease progresses in *Ndufs4*(KO)’s, animals can display intermittent/transient improvement of symptoms. Here, we report the age at which animals first present symptom which persist for two or more consecutive days.

#### Survival:

The same euthanasia criteria were applied to both the *Ndufs4*(−/−) and *Gpx4* conditional knockout models. Animals were humanely euthanized when they had lost 20% of their body weight from maximum (measured twice consecutively), presented with acute motility or neurologic symptoms perceived to impair ability to access to food or water (immobility, prostrate posture, etc), or if they were otherwise moribund in appearance (inactive and dehydrated, etc).

### Gpx4 conditional knockout mice

G*px4* floxed allele animals (*Gpx4*(fl/fl)) were obtained from Jackson laboratories (strain 027964). In these animals, exons 2–4 of *Gpx4* are flanked by loxP sites, allowing for conditional or inducible knockout by expression of Cre recombinase. Rosa26CreERT2, which carry a pan-expression tamoxifen inducible Cre cassette, were also purchased from Jackson laboratories (strain 008463). These two lines were bred to generate *Gpx4*(fl/fl)/Rosa26CreERT2(+/+) line. Genotyping of Rosa26CreERT and *Gpx4*(fl/fl) was performed as detailed in Supplemental Files 1 (‘Gpx4 floxed Genotyping Protocol’)and Supplemental File 2 (‘Rosa26CreERT2 Genotyping Protocol’). Tamoxifen treatment for Cre induction occurred on P25-P28; animals were injected with 60 µg tamoxifen/g mouse/day on P25, P26, and P27, and 60 µg tamoxifen/g mouse on P28 (see also Results). Animals treated with vatiquinone or vehicle for vatiquinone were injected with the drug starting at P21, prior to tamoxifen driven induction of Cre expression. On the days when both tamoxifen and vatiquinone (or vehicle for vatiquinone) were both provided, they were provided as a mixture to minimize the number and total volume of injections. Ataxia was scored by visual assessment with any overtly uncoordinated gait or overt reduction in overall movement leading to a score of ataxia.

As previously reported for this model (1, 12), tamoxifen injections elicited disease in all Gpx4(fl/fl)/Rosa26CreERT2 mice but in none of the Gpx4(WT/WT) or Gpx4(WT/fl) animals (tamoxifen treatment details below). No symptoms were ever observed in Gpx4(fl/fl)/Rosa26CreERT2 mice that were injected with oil only (rather than tamoxifen) or not injected, as has been previously reported.

### Ndufs4*(−/−) mice*

*Ndufs4*(+/–)mice (originally obtained from the Palmiter laboratory, University of Washington, Seattle, Washington, USA; available through The Jackson Laboratory, strain 027058) were bred to produce *Ndufs4*(−/−) offspring. Mice were weaned at 20–21 days of age and *Ndufs4*(−/−) animals were housed with control littermates for warmth and stimulation. Mice were weighed and health was assessed a minimum of 3 times per week, daily on days when injected. Wetted chow in a dish on the bottom of each cage, and in-cage water bottles, were provided to cages housing *Ndufs4*(−/−) mice following onset of symptoms so that food and water accessibility was not a limiting factor for disease progression and survival. Vatiquinone and vehicle treatments started at P21, weaning. Animals were humanely euthanized when they had lost 20% of their body weight from maximum (measured twice consecutively), on presentation with acute motility or neurologic symptoms perceived to impair ability to access to food or water (immobility, prostrate posture, etc) or if they were otherwise moribund in appearance (inactive and dehydrated, etc). As previously reported, the *Ndufs4* deletion is fully recessive: heterozygosity results in no reported phenotypes and no detectable defects in ETC CI activity. Accordingly, “control” cohorts include *Ndufs4*(+/−) and *Ndufs4*(+/+) mice.

### Longitudinal assessments of disease

**Figure 2:**
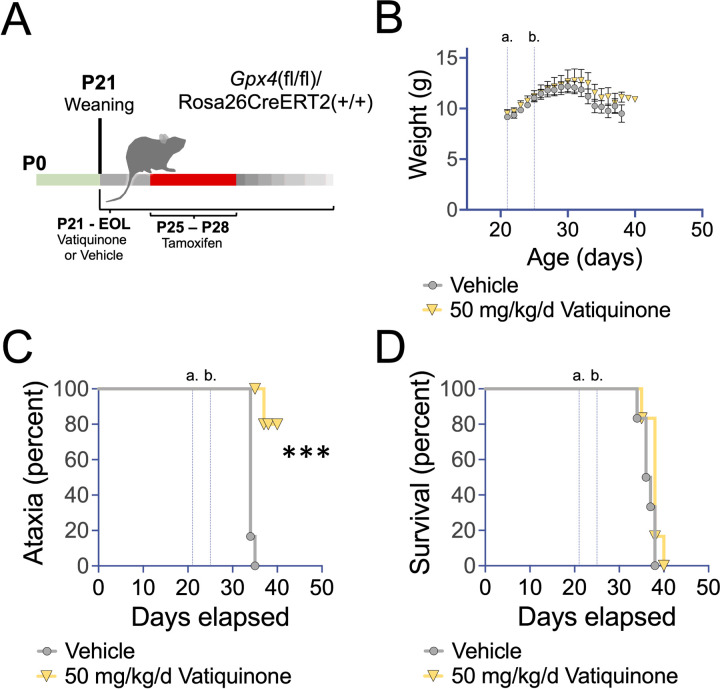
Vatiquinone delays ataxia but not death in an inducible *Gpx4* knockout model. (A) Schematic of studies in the *Gpx4*(fl/fl)/Rosa26CreERT2(+/+) inducible model for *Gpx4* deficiency. Treatment with vatiquinone (50 mg/kg/day) or vehicle (see Methods) by IP injection began at weaning, P21, and continued until end of life criteria were met. Tamoxifen was administered from P25-P28, provided in one injection with vatiquinone or vehicle (see Methods). (B) Weight curves of *Gpx4*(fl/fl)/Rosa26CreERT2(+/+) treated with tamoxifen at P25-P28 and vatiquinone or vehicle from P21. Vertical lines indicate weaning and start of vatiquinone or vehicle treatment (a.) and P25, the beginning of Cre induction by tamoxifen (b.). Data are shown as mean with error bars displaying standard error of the mean. There were no significant differences in weight by treatment group at any age. (C) Onset of ataxia by visual assessment (see Methods). Vatiquinone treated inducible *Gpx4* knockout animals showed a statistically significant delay in the appearance of ataxia (***p<0.0005 by log-rank test). Vertical lines show weaning (a.) and first day of tamoxifen treatment (b.) as in (B). (D) Survival of inducible *Gpx4* deficient animals. Vertical lines show weaning (a.) and first day of tamoxifen treatment (b.) as in (B). Vatiquinone did not alter survival in inducible *Gpx4* deficient mice. (B-C) n=6 animals per group.

**Figure 3:**
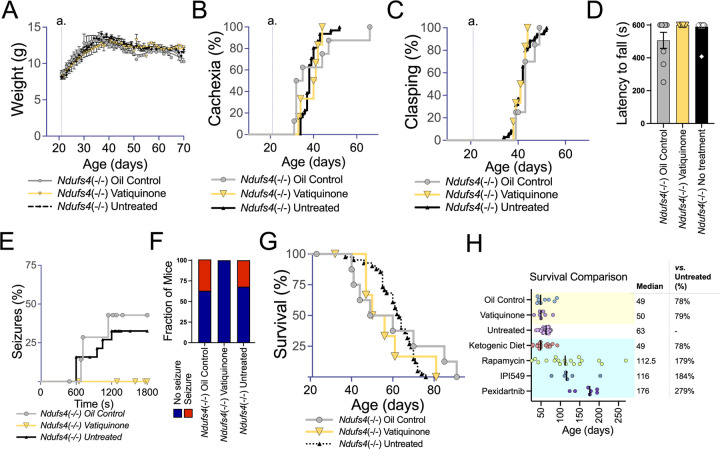
Vatiquinone does not impact overall disease or survival but may prevent seizures in the *Ndufs4*(−/−)model of Leigh syndrome. (A) Weights of untreated, vatiquinone treated, or vehicle (oil) only treated *Ndufs4*(−/−) mice. Data are shown as mean with standard error of the mean. No significant differences in weight were observed at any age. Weaning and the start of treatments occur at P21 (line a.). (B) Onset of cachexia in untreated *Ndufs4*(−/−) mice and *Ndufs4*(−/−) mice treated with vatiquinone or vehicle (oil) only. Cachexia onset is defined as the age in each individual animal when weight peaks and progressive weight loss begins (see (A) for overall weight trajectory for *Ndufs4*(−/−) mice). Weaning and the start of treatments occur at P21 (line a.). Vatiquinone had no significant effect when compared to either vehicle (oil) or untreated cohorts. (C) Onset of forelimb clasping in untreated *Ndufs4*(−/−) mice and *Ndufs4*(−/−) mice treated with vatiquinone or vehicle (oil) only. Vatiquinone had no significant effect when compared to either vehicle (oil) or untreated cohorts. Weaning and the start of treatments occur at P21 (line a.). (D) Rotarod performance at P30, reported as the best of three trials with a maximum trial time of 600 seconds (see Methods). Treatment groups did not significantly differ, but there is no overt defect in performance at this age; rotarod trials from this age are used to assess seizure frequency (see (E-F)). (E) Seizure incidence as a function of overall time on the rotarod at P30 in untreated *Ndufs4*(−/−) mice and *Ndufs4*(−/−) mice treated with vatiquinone or vehicle (oil) only. Differences did not reach statistical significance (log-rank test) in these limited cohorts, but vatiquinone treated animals did not present with seizures in this assay. (F) Overall seizure incidence in the P30 rotarod assay (panels (D-E)). Differences did not reach statistical significance in these limited cohorts (Fisher’s exact test p=0.156 vatiquinone vs summed control treatments), but no seizures were observed in vatiquinone treated animals. (D-F) n=16 (untreated), 8 (vehicle treated), and 6 (vatiquinone treated). (G) Survival of untreated *Ndufs4*(−/−) mice and *Ndufs4*(−/−) mice treated with vatiquinone or vehicle (oil) only. No significant differences were observed between treatment groups (log-rank test). (H) Survival data from animals in this study (green highlighted region of plot) and additional representative results from other small-molecule intervention trials performed by our group including ketogenic diet (9), 8 mg/kg/day rapamycin by IP injection (8), 100 mg/kg/day IPI-549 orally in chow (11), and 300 mg/kg/day pexidartinib orally in chow (11). IPI549 and pexidartinib studies occurred partly in tandem with the vatiquinone trials, and the untreated *Ndufs4*(−/−) mouse cohort spanned these trials. Rapamycin, IPI-549, and pexidartinib were found to significantly increase survival, while ketogenic diet had no significant effect (see cited literature for details). Vatiquinone similarly has no significant effect on survival in the *Ndufs4*(−/−). (A-G) n=9 oil treated and 7 vatiquinone treated *Ndufs4*(−/−) animals per dataset.

Forelimb clasping, a sign of neurologic disease progression, was assessed by visual scoring, as previously described (8–11). As disease progresses in *Ndufs4*(KO)’s animals display intermittent/transient improvement of symptoms. Here, we simply report whether animals ever presented the symptom for two or more consecutive days (this criterion to minimize spurious reporting). Cachexia onset (Fig. 2 and Fig. 3) is defined as the day of life when an individual animal’s weight peaks and progressive weight loss starts, as in our prior studies (9–11, 13).

### Drugs for injection

Vatiquinone stock was prepared by dissolving vatiquinone in sunflower seed oil to a final concentration of 5 µg/uL (w/v), passing through a 0.22 micron sterile filter, aliquoting, and storing aliquots at −20°C (and protected from light) until use. Vatiquinone treated mice were injected with 10 µL/g of this solution (10 µL/g body weight = 50 µg vatiquinone/g body weight), and vehicle treated mice injected with 10 µL/g of sterile sunflower seed oil only. Injected solutions were warmed to 25–37°C prior to injection.

Tamoxifen was prepared at 6 mg/mL in 95% (by volume) sunflower seed oil/5% (by volume) ethanol and stored in sterile (0.22 micron filtered) aliquots.

#### Cre induction regimen:

On P25-28, Gpx4(fl/fl)/Rosa26CreERT2 animals were injected with 10 µL/g for a dose of 60 µg/g/day. On P29, animals this was diluted 1:1 with 95% sunflower seed oil/5% ethanol for a dose of 30 µg/g (half dose) on the final induction day. In animals treated with vatiquinone and tamoxifen, the injection solution was prepared with both 5 µg/uL vatiquinone and 6 mg/mL (or 3 mg/mL on the last day) tamoxifen in the same mixture, allowing for administration of both compounds in the same injection. Control (no vatiquinone) animals were provided matching vehicle injections on each day.

### Rotarod and rotarod-induced seizures

Mouse rotarod performance was assessed at P30, with seizure incidence noted. Rotarod was performed using a Med Associates ENV-571M single-lane rotarod. Assays were performed by placing animals onto an already rotating rod and timing latency to fall. Rotation was set to constant 6 rpm (controlled using Med Associates software). Maximum time of each trial was 10 min. For each assay, three trials were performed, with a minimum of 5 min between trials. The greatest latency to fall of the three trials was used for comparisons.

Mice were monitored during each trial for seizure activity. Seizures were identified by any of the symptoms on the Racine behavioral scale or Pinel and Rovner scales, but abnormal oroalimentary movements (dropping of the jaw repeatedly, atypical gnawing or chewing movements), and repeat head nodding were not considered ‘definitive’ for seizure activity and did not lead to trial halt or seizure scoring. Anterior limb clonus (twitching/jumping), dorsal extension/rearing, loss of balance and violent falling (observed when mouse was not on the rotarod), violently running/jumping were considered definitive of seizures and resulted in both a halt to trials that day and recording of a seizure incident. No attempt was made to score seizure severity for these studies – we assessed only presence vs absence.

## Results

### Vatiquinone suppresses RSL3 and BSO/Fe(III)C induced cell death

To establish the efficacy of our commercially obtained vatiquinone (MedKoo, see Methods) in cultured cells, we treated human neonatal dermal fibroblasts (HDFs) with RSL3 in the presence or absence of vatiquinone (see Methods). RSL3 is an inhibitor of the mitochondrial antioxidant enzyme glutathione peroxidase 4, Gpx4, and a strong inducer of ferroptotic cell death via this inhibition (7, 14). Vatiquinone has been shown potently suppress RSL3-induced ferroptosis in mitochondrial disease patient cell lines via inhibition of 15-lipoxygenase (15-LO)(see Discussion)(7). In HDFs, 0.2 µM and 2 µM RSL3 led to approximately 20% and 50% cell death, respectively, by 24 hours of exposure and RSL3-induced cell death was fully suppressed by 500 nM vatiquinone, in line with previous reports using vatiquinone provided by BioElectron (Fig. 1A–C) (7).

Vatiquinone has also been shown to prevent cell death resulting from treatment with L-buthionine-(S,R)-sulfoximine (BSO) and Fe(III)Citrate (Fe(III)C). BSO is an irreversible inhibitor of γ-glutamylcysteine synthetase (γ-GCS) which drives depletion of glutathione via inhibition of synthesis. When cells are treated with BSO and provided with excess labile iron, which can be added in the form of Fe(III)Citrate, ferroptosis is induced (7, 15). Indirect inhibition of Gpx4 via glutathione depletion has been shown to mediate ferroptosis induction by BSO/Fe(III)C. As with RSL3, we found that vatiquinone robustly prevents BSO/Fe(III)C induced cell death in HDFs (Fig. 1D–F).

### Vatiquinone has no impact on paraquat induced cell death

Vatiquinone was identified in a screen using cell viability following glutathione depletion, and RSL3 is an inhibitor of the glutathione peroxidase Gpx4, but vatiquinone is frequently referred to as a general antioxidant (16). Vatiquinone has been described as having ‘up to 10,000 times [the] antioxidant potency’ of CoQ_10_ (17), but data assessing the effects of vatiquinone in other forms of oxidative stress are limited. To determine whether the benefits of vatiquinone extend to forms of oxidative stress beyond ferroptosis, as frequently suggested, we next treated HDFs with paraquat. Paraquat (N, N′-dimethyl-4,4′-bipyridinium dichloride, also known as methyl viologen) is a toxin that generates high levels of reactive oxygen species (ROS) at mitochondrial ETC CI (18, 19). In contrast to RSL3 and BSO/Fe(III)C, vatiquinone had no impact on paraquat induced cell death (Fig. 1G–I).

#### Vatiquinone may delay onset of ataxia, but does not extend survival, in Gpx4 deficient mice

After confirming the robust benefits of vatiquinone in cultured cells treated with RSL3 and BSO/Fe(III)C, we next sought to establish whether clinically relevant doses of vatiquinone can attenuate disease resulting from Gpx4 loss *in vivo*. To test this, we used an inducible *Gpx4* knockout mouse line by crossing mice carrying an allele of *Gpx4* where exons 2–4 are flanked by loxP sites (Jackson laboratories strain 027964) and a tamoxifen-inducible, whole-body expressed, Cre recombinase (Rosa26CreERT2, Jackson laboratories strain 008463, see Methods). This approach has previously been used to study the impact of *Gpx4* loss *in vivo* (12), with postnatal loss of *Gpx4* causing mitochondrial dysfunction, mitochondrial oxidative damage, apoptosis, neurodegeneration, and death within 2 weeks (see Discussion)

Using animals homozygous for both alleles, we treated animals with either vatiquinone or vehicle solution starting at weaning, postnatal day 21 (P21), by daily IP injection; animals were also treated with tamoxifen from P25-28 to induce Cre expression (Fig. 2A, Methods). As previously reported, induction of Cre in these mice led to rapid progressive weight loss, onset of ataxia, and mortality within about two weeks (Fig. 2B–D).

Mice were treated with vehicle (oil) only or 50 µg/kg body weight/day (50 mg/kg body weight/day) vatiquinone. This dose was based on that used for clinical trials and efficacy *in vitro* (see Table 2). 50 mg/kg/day vatiquinone had no effect on survival or weight loss in *Gpx4* deficient mice but, interestingly, appeared to delay the onset of ataxia.

#### Vatiquinone has no effect on overall disease progression in the Ndufs4(−/−) mouse model of Leigh syndrome, but may reduce seizures

To test the efficacy of vatiquinone in the context of Leigh syndrome, we used the *Ndufs4*(−/−) mouse model of the disease. *Ndufs4*(−/−) animals were treated with 50 mg/kg/day vatiquinone or vehicle solution only starting at weaning, P21, by daily IP injection (see Methods). This approach has been effective in other pharmacologic studies in this model, including our own prior work (see Discussion and Fig. 3H). Onset of weight loss (cachexia) and overall progressive weight decline from disease onset were not impacted by either vehicle (oil) treatment or by vatiquinone (Fig. 3A–B). Similarly, neither the vehicle nor vatiquinone impacted the age of onset of forelimb clasping, a visually scored symptom of neurologic disease or mortality (Fig. 3C)

*Ndufs4*(−/−) performance on a rotarod assay is not significantly impaired at P30 (Fig. 3D), but the rotarod assay at this age can be used to assess exercise-induced seizure incidence (9). Overall exercise-induced seizure incidence and time to seizure were the same in oil (vehicle) treated and untreated *Ndufs4*(−/−) mice, but vatiquinone appeared to suppress exercise-induced seizures at this age (Fig. 3E–F, see Discussion regarding statistical significance).

Survival of *Ndufs4*(−/−) animals was not significantly impacted by either the vehicle (oil) or by 50 mg/kg/day vatiquinone (Fig. 3G–H).

## Discussion

### Vatiquinone – a potent drug for a specific target

Vatiquinone/EPI-743 was first identified in a small molecule screen for compounds which prevent cell death caused by the glutathione synthesis inhibitor L-buthionine-(S,R)-sulfoximine (BSO) (16). These benefits have recently been attributed to inhibition of 15-lipoxygenase (15-LO), which generates the oxidized lipid product 15-hydroxyeicosatetraenoic acid when glutathione levels are low or GPX4 is inactivated (7). Here, we’ve been able to reproduce the effects of vatiquinone in preventing cell death from either GPX-4 inhibition or glutathione depletion with iron overload (see Fig. 1). In these specific conditions, vatiquinone appears to be potent, providing robust benefits.

In contrast, we find that vatiquinone has no impact on paraquat-induced cell death, indicating that vatiquinone is not effective as a general antioxidant or mitochondrial antioxidant. These cell-based findings suggest that vatiquinone benefits are probably limited to perturbations of the GPX4/15-LO axis. Our data do not support the notion that vatiquinone would benefit conditions defined by generalized mitochondrial oxidative stress, unless 15-LO mediated cell death has been observed. That scenario may include FA, genetic defects in GPX4, and other factors involved glutathione redox or iron homeostasis, but no current evidence supports the idea that 15-LO is involved in the pathobiology of mitochondrial diseases as a group or Leigh syndrome in particular.

#### GPX4 *deficiency*

Defects in GPX4 can cause Sedaghatian-type spinal metaphyseal dysplasia (SSMD), a severe genetic disease characterized by metaphyseal chondrodysplasia, cardiovascular disease, and neurologic defects (1). SSMD mutations have been shown to impact enzymatic activity of GPX4. The *Gpx4* conditional knockout mouse model has been used to study disease arising from GPX4 defects, with Cre leading to the accumulation of mitochondrial (but not whole-cell) 4-hydroxynonenal (4-HNE, a product of ROS damage), a reduction in mitochondrial complex I activity and ATP generation capacity, overt cell death (including neurons), and animal death within 5–15 days (12). Neuron specific knockout leads to a nearly identical rate of death (20). It was claimed that high dose vitamin E delayed disease, but the extremely small effect (1 day delay in mortality) and lack of any statistical comparisons does not support that conclusion. An examination of the data indicates vitamin E is not effective against GPX4 deficiency.

Given the robust benefits of vatiquinone in the setting of *Gpx4* inhibition in cultured cells, we found it surprising that the compound had no effect on survival in the inducible knockout mouse. One possibility is that total loss of Gpx4 has effects beyond induction of cell death via 15-LO. Gpx4 loss in mice does appear more severe than SSMD-causing GPX4 mutations in humans as loss in mice causes embryonic lethality or rapid death after postnatal deletion. Accordingly, it is unclear whether *Gpx4* deficient mice are a useful disease model for SSMD.

Intriguingly, ataxia onset was significantly attenuated by vatiquinone in this cohort. These observations will need to be validated, and histologic analysis of neuronal loss performed, to confirm these findings. Future studies using the neuron-specific *Gpx4* deficient model may provide additional insight into the role of 15-LO mediated neuronal death or dysfunction in neurologic sequelae resulting from *Gpx4* loss.

### Leigh syndrome

A number of clinical trials focus on vatiquinone in Leigh syndrome (see below, Table 1). In contrast to SSMD, a robust a well-established mammalian model for Leigh syndrome is available, the *Ndufs4*(−/−) mouse. This animal shares a causal genetic defect with a subset of human Leigh syndrome patients (complete loss of NDUFS4) and displays the major sequelae of the human disease. In particular, the animal model develops progressive, symmetric, neuroinflammatory brain lesions, a defining clinical feature of Leigh syndrome. Multiple pre-clinical interventions have been found to significantly attenuate disease in the *Ndufs4*(−/−). Among these are inhibition of mTOR or PI3Kγ, chronic mild hypoxia at 11% oxygen, which prevents and reverse symptoms, and the CSF1R inhibitor pexidartinib, which prevents disease in the *Ndufs4*(−/−) but is itself toxic in chronic use. Against this backdrop of effective interventions, 50 mg/kg/day vatiquinone had no impact on overall disease onset and progression (see Fig. 3H).

### Seizures

Exercise-induced seizures occur in *Ndufs4*(−/−) at P30 are reduced by therapies including rapamycin and a ketogenic diet (9). Similar to dietary ketosis, vatiquinone failed to alter overall disease course or extend survival but appears, in our limited cohort, to suppress these seizures (Fig. 3E–F). This is intriguing in light of the MIT-E trial, which failed to achieve primary endpoints but, according to a press release, significantly reduced seizure incidence in the subset of patients with Leigh syndrome (NCT04378075, see Table 1)(7) (June 29, 2023 press release from PTC Therapeutics). Together, clinical and pre-clinical data seem to indicate that 15-LO may be a viable therapeutic target for seizures in Leigh syndrome.

Any mechanistic link between 15-LO and seizures is not immediately clear. Furthermore, our limited supply of vatiquinone allowed (see Methods) did not allow for animal numbers sufficient to reach statistical significance; our observations are interesting, but highly preliminary. Follow-up studies should consider the impact of vatiquinone in the GAD2-specific *Ndufs4*(−/−) model, which presents with fatal mitochondrial epilepsy but not progressive neuroinflammatory CNS lesions (21, 22). This will enable study of seizures in the absence of other, complicating, disease sequelae.

### Clinical significance and ethical perspectives

In the US alone, there have been 19 clinical trials of vatiquinone. Of these, 13 target mitochondrial disease, with six targeting Leigh syndrome either as a component of mitochondrial disease overall (NCT05218655, NCT04378075, NCT01370447, NCT01642056) or as a specific clinical indication (NCT02352896, NCT01721733)(see Table 1). The earliest of these trials dates to 2013. However, as of this writing, results have only been posted to trials.gov for one study (NCT01642056). This trial targeted children with mitochondrial disease and failed to achieve its primary outcomes. Press reports indicate that four additional studies have failed to achieve primary outcomes.

In smaller studies, some promising results have been reported, but these have lacked rigor and sufficient sample size for confident analysis. Three case-reports testing vatiquinone in Leigh syndrome with ten, four, and one patient, respectively, found vatiquinone to be well-tolerated (16, 23, 24). These studies report some indications suggesting the drug might modestly impact disease progression, but the cohorts are too small, clinical outcomes too unrefined, and benefits to weak for confidence. Additionally, the initial reports from 2012 lack any published follow-up.

Clinical evidence supporting the efficacy of vatiquinone in Leigh syndrome, and mitochondrial disease broadly, has failed to materialize. Nevertheless, anecdotal evidence persists, and our experiences with patient groups indicate that many believe this compound represents a major advance in treating mitochondrial disease. Disease course is known to be highly variable in mitochondrial disease, but ebbs in clinical progression are often viewed as linked to any therapy being provided. Misrepresentation of vatiquinone in the scientific literature (for example, as an ‘antioxidant’, ‘scavenger’, or compound that ‘generates glutathione’, and statements that ‘EPI-743 works by improving the regulation of cellular energy metabolism’) exacerbate these issues (citations intentionally omitted).

The failure of vatiquinone to alter disease course in the *Ndufs4*(−/−) model has implications for any future trials. These findings also raise questions regarding the ethics of testing experimental compounds in rare disease patients when the mechanism of action is unknown (15-LO was only recently identified as the target of vatiquinone) and they have not been tested in relevant preclinical animal models. Our observations highlight the lack of reported results from relevant trials (see Table 1). Mitochondrial disease progression is highly varied; off-label, small cohort, and uncontrolled trials can easily result in overly optimistic views of a candidate agent. Speedy reporting of results from controlled trials, whether positive or negative, should be a priority, as should selection of the most promising candidate therapeutic approaches. Populating trials is difficult in rare diseases; testing agents with no proven efficacy results in missed opportunities for the patients and for progress in understanding and treating mitochondrial disease.

Since the first trial of vatiquinone started in 2012, significant progress has been made in identifying therapeutic targets in the mouse model. Most notably chronic mild hypoxia and immune targeting show significant promise (8, 10, 25–30). We believe that strategies showing efficacy in a robust animal models should be prioritized over those lacking *in vivo* evidence, and that agents with known mechanism of action should be prioritized over those whose targets are unknown. Finally, the clinical data suggests that target population should be more carefully selected according to the precise mode of action of the drug. Preliminary clinical and pre-clinical evidence suggests vatiquinone may benefits mitochondrial seizures, and cell-based data indicate vatiquinone should benefit settings of increased 15-LO mediated cell death, but no data support the notion that vatiquinone should be effective in mitochondrial diseases as a broad clinical group.

## Supplementary Material

Supplement 1 Tables 1 and 2 are available in the Supplementary Files section.

## Data Availability

All data are available within the article.
