# Supplementary material for: Evaluating the efficacy of vatiquinone in preclinical models of mitochondrial disease
Source: Res Sq. 2024 Jun 3:rs.3.rs-4202689. Preprint. [Version 1] doi: 10.21203/rs.3.rs-4202689/v1 (PMC11177993; doi:10.21203/rs.3.rs-4202689/v1)
Supplement: Supplement 1 — Tables 1 and 2 are available in the Supplementary Files section. [file NIHPPrs4202689v1-supplement-1.pdf]

Supplementary Files

This is a list of supplementary files associated with this preprint. Click to download.

- Table1.docx
- Table2.docx
